# Use of diffusion magnetic resonance imaging to correlate the developmental changes in grape berry tissue structure with water diffusion patterns

**DOI:** 10.1186/1746-4811-10-35

**Published:** 2014-11-04

**Authors:** Ryan J Dean, Timothy Stait-Gardner, Simon J Clarke, Suzy Y Rogiers, Gabriele Bobek, William S Price

**Affiliations:** Nanoscale Organisation and Dynamics Group, University of Western Sydney, Penrith, NSW 2751 Australia; National Wine & Grape Industry Centre, Charles Sturt University, Locked Bag 588, Wagga Wagga, New South Wales, 2678 Australia; New South Wales Department of Primary Industries, Locked Bag 588, Wagga Wagga, New South Wales, 2678 Australia; School of Medicine, University of Western Sydney, Penrith, NSW 2751 Australia

**Keywords:** Development, Diffusion anisotropy, Diffusion tensor imaging, Grape berry, Nucellus, Nuclear magnetic resonance imaging, Olive, Seeds, Striation patterns, *Vitis vinifera*

## Abstract

**Background:**

Over the course of grape berry development, the tissues of the berry undergo numerous morphological transformations in response to processes such as water and solute accumulation and cell division, growth and senescence. These transformations are expected to produce changes to the diffusion of water through these tissues detectable using diffusion magnetic resonance imaging (MRI). To assess this non-invasive technique diffusion was examined over the course of grape berry development, and in plant tissues with contrasting oil content.

**Results:**

In this study, the fruit of *Vitis vinfera* L*.* cv. Semillon at seven different stages of berry development, from four weeks post-anthesis to over-ripe, were imaged using diffusion tensor and transverse relaxation MRI acquisition protocols. Variations in diffusive motion between these stages of development were then linked to known events in the morphological development of the grape berry. Within the inner mesocarp of the berry, preferential directions of diffusion became increasingly apparent as immature berries increased in size and then declined as berries progressed through the ripening and senescence phases. Transverse relaxation images showed radial striation patterns throughout the sub-tissue, initiating at the septum and vascular systems located at the centre of the berry, and terminating at the boundary between the inner and outer mesocarp. This study confirms that these radial patterns are due to bands of cells of alternating width that extend across the inner mesocarp. Preferential directions of diffusion were also noted in young grape seed nucelli prior to their dehydration. These observations point towards a strong association between patterns of diffusion within grape berries and the underlying tissue structures across berry development. A diffusion tensor image of a post-harvest olive demonstrated that the technique is applicable to tissues with high oil content.

**Conclusion:**

This study demonstrates that diffusion MRI is a powerful and information rich technique for probing the internal microstructure of plant tissues. It was shown that macroscopic diffusion anisotropy patterns correlate with the microstructure of the major pericarp tissues of cv. Semillon grape berries, and that changes in grape berry tissue structure during berry development can be observed.

## Background

Magnetic resonance imaging (MRI) is a powerful tool for investigating biological systems; it is non-invasive, does not use ionising radiation and can draw on a large variety of image contrasts [1, 2]. For example, in the field of viticulture, MRI has demonstrated its ability to produce informative proton (^1^H) density and transverse magnetic relaxation images of the internal structure of the grape berry [3, 4]. Despite the good spatial resolution of the images from the aforementioned studies, it is possible to extract further microstructural (i.e. beyond the resolution limit of an MRI spectrometer) information from a grape berry. This can be achieved using diffusion MRI techniques to observe the distribution of diffusing molecules as they probe the edges of grape berry tissue microstructures. Diffusion MRI has been used to study a variety of different botanical species; for example, maize stems [5, 6], barley seeds [7], carrot roots [8], celery stems [9, 10] and asparagus stems [11, 12].

A recent study has produced diffusion tensor (DT) images (i.e. three-dimensional diffusion images) of the grape berry [13]. While the primary focus of this study was the grape berry vascular system, the orientation of anisotropic diffusion of the berry pericarp was also visible. However, the anisotropy of these observed diffusion patterns did not appear to be related to the known pericarp tissue structure. This is contrary to the commonly shared expectation that anisotropic diffusion should correlate with the orientation of organised tissue structures [14, 15].

The objective of the current study was to verify whether diffusion anisotropy features can reflect the relative size, orientation and organisation of the cells that constitute the different tissues of the grape berry pericarp. As previous MRI studies of grape berry tissue structure have focussed on grape berries at single time-points, the second objective of the study was to examine structural changes across grape berry development via diffusion and relaxation MRI. Additional experiments using a postharvest olive as a test sample demonstrated that the diffusion and relaxation MRI techniques are applicable to the study tissue structure of fruits with high oil content.

Since the interpretation of the results of the current investigation require some understanding of relaxation and diffusion MRI, the salient points of both imaging techniques are presented in the following section. More detailed coverage of these topics is available elsewhere [14, 16].

### Transverse relaxation and diffusion MRI theory

MRI image contrast depends on the pulse sequence used to weight the measured magnetic resonance (MR) signal. The two forms of MR image contrast used in the current study were the rate of transverse magnetic relaxation and translational self-diffusion.

The transverse relaxation (i.e., spin-spin relaxation) of magnetisation relates to the rate at which the transverse component of the nuclear spin magnetisation vanishes after the system has been excited by the application of electromagnetic radio frequency pulses [17]. The relaxation rate is mainly related to the reorientational motions of the molecule (or that part of the molecule containing the nuclear spin) with slowly moving nuclei (i.e. being in a solid or more viscous environment) relaxing more quickly. Unbound paramagnetic ions (e.g. iron and manganese ions), can also increase the rate at which spins relax. Transverse relaxation weighted images are commonly measured using a Carr-Purcell-Meiboom-Gill (CPMG) MRI pulse sequence [18, 19].

Translational self-diffusion, on the other hand, is the random, thermal motion of freely moving molecules such as water. This is distinct from mutual diffusion (i.e. molecular motion due to the presence of a chemical potential). For molecules in bulk solution, translational self-diffusion (henceforth referred to as ‘diffusion’) can be described using the scalar diffusion coefficient (*D*). For water molecules in bulk solution at standard room temperature, *D* ≈ 2.29 × 10^-9^ m^2^ s^-1^ [20]. The root mean square displacement (RMSD) of a species diffusing in one dimension is given by
1

where Δ is the timescale of the measurement [21]. Water molecules in bulk solution at room temperature diffusing over a period of 50 ms have a RMSD of ~15 μm (i.e. the length scale of eukaryotic cell compartments [22]). The pulsed gradient spin-echo (PGSE) diffusion MRI sequence measures the displacement of the diffusing molecules along the direction of the applied magnetic field gradient used in the sequence [14, 23, 24]. The intensity of the diffusion weighted MR signal is given by;
2

where *S* is the diffusion weighted MR signal intensity, *S*_0_ is the diffusion unweighted MR signal intensity, *E* is the MR signal attenuation, *γ* is the gyromagnetic ratio of the nuclei of interest, *g* is the diffusion magnetic gradient strength, *δ* is the length of the applied magnetic field gradient pulse, and Δ is the diffusion period. The diffusion weighting of the pulse sequence can be summarised as a scalar factor, *b* [14, 23], where
3

**Figure 1:**
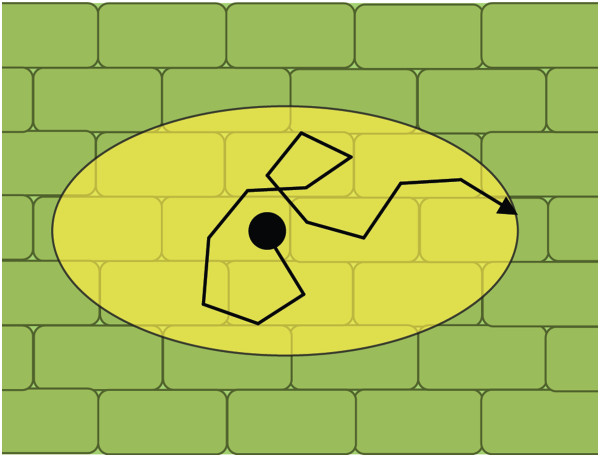
**Anisotropic water diffusion due to diffusive restriction (two dimensional representation).** Here water molecules (●) encounter cellular boundaries as they diffuse. As there are more cellular boundaries on the longitudinal axis than the lateral axis, water displacement along the longitudinal axis is reduced relative to the lateral axis (represented by the yellow ellipse).

When diffusing molecules encounter physical obstructions (e.g. cellular boundaries) their molecular displacement is restricted [14, 25]. Consequently, if *D* is computed from a measured RMSD using Eq. (1) an ‘apparent’ diffusion coefficient (ADC), which is equal to or smaller than the bulk *D*, will be determined. The ADC of water measured across a span of cells is dependent on various morphological features, such as cell size and orientation, and the permeability of the cellular membrane [26]. As such, the measured ADC will depend upon the direction of measurement. If molecular mobility is equal in all directions, the apparent diffusion is isotropic and a single ADC is sufficient to characterise diffusion. Conversely, if mobility is not equal in all directions due to the arrangement of microscopic tissue structures, the apparent diffusion is anisotropic (Figure 1). The direction of greatest diffusion mobility (i.e. the direction of least diffusive restriction) can be indicated with a diffusion vector. In the case of anisotropic diffusion, the directional dependence of diffusion can be included in Eq. (2) by replacing *D* with a diffusion tensor, **D** [24, 27]. In order to reconstruct **D**, PGSE measurements along six or more non-collinear directions are performed. This form of MRI is termed diffusion tensor imaging (DTI).

## Methods

### Grape berry growing conditions

*Vitis vinifera* L. cv. Semillon (clone DA16162, own roots) berries were obtained from 18 grapevines grown in a glasshouse at the National Wine & Grape Industry Centre, Wagga Wagga, NSW, Australia. The plants were maintained in 35 L pots containing sandy loam. In late winter each plant was pruned to a pair of two-bud spurs and brought into the glasshouse to commence a fifth season of vegetative growth. Average daily maximum/minimum temperature and relative humidity in the glasshouse across the growing season were 30/17°C and 75/40%, respectively. Each plant was watered beyond field capacity four times per day via a pair of drip emitters. Diluted fertiliser (Megamix Plus, Rutec Pty Ltd, Tamworth, Australia, 20 mL per plant) was applied fortnightly to the soil and wettable sulphur/tribasic copper sulphate was sprayed on the shoots. The shoots were trained vertically and pruned to one inflorescence each approximately six weeks after bud burst. The date of bud burst was assessed on each bud [28] and the date of flowering (approximately 100% capfall) was assessed on each inflorescence.

### MRI hardware and software

All ^1^H MRI was performed on a 500 MHz (11.7 T) wide-bore nuclear magnetic resonance spectrometer (AVANCE II; Bruker Biospin Co., Ltd., Germany), equipped with triple axis gradients capable of generating 1.5 T m^-1^ magnetic field gradients. Each imaging experiment employed a 30 mm birdcage radio frequency coil insert. Standard Bruker sequences were used (see Experimental procedures). Data acquisition, post-processing and imaging was controlled from a computer terminal running a Linux operating system, using ParaVision (5.1; Bruker Biospin Co., Ltd.). Image analysis and additional post-processing was performed on a separate computer terminal running Microsoft Windows 7. To analyse the images, the raw MRI data were imported into MATLAB (8.0.0.783, the MathWorks, USA) for analysis using in-house programs.

### Experimental procedures

Berries were sampled fortnightly for imaging, beginning four weeks after flowering and ending three months later. To minimise developmental variability at each sampling event, grape bunches were assigned to classes according to flowering date (28, 41, 55, 70, 84, 95 and 109 days after flowering (DAF) respectively). Seven bunches were randomly chosen per sampling event. Each sample consisted of the distal portion of the bunch (approximately 10 berries), which were obtained by cutting the rachis while it was momentarily submersed in tap water. The detached, distal portion of the bunch was wrapped in a moist paper towel, placed in a zip-lock bag and shipped overnight to the Biomedical Magnetic Resonance Facility located at the University of Western Sydney, Campbelltown, NSW, Australia.

Three berries were examined per age class (for a total of twenty-one grape berries) to monitor changes in the diffusion pattern across the berry over the course of its development. Berries of an average size (with respect to the bunch) were cut 3 to 4 mm above the pedicel. All MRI protocols were completed within 11 hours of berry detachment. After imaging, the berry was dried at 70°C and then weighed. All grape bunches were stored at 4°C while not in use. Once selected for imaging, a grape berry had approximately 30 minutes to equilibrate to room temperature, maintained at 22 ± 0.1°C, during the spectrometer setup and calibration stages. The interior temperature of the spectrometer was also maintained at 22 ± 0.1°C. The total soluble solids (°Brix) was estimated for each grape berry imaged, based on the average of three refractormeter (PAL-1, Atago Co., Ltd., Tokyo, Japan) readings obtained from berries of the same bunch as the berry imaged.

Each berry was imaged using the MRI sequences described below. Pulse sequence parameters include the slice thickness (THK); the matrix size (MTX), which indicates the number of voxels (i.e. volume elements) which comprise the image; the repetition time (TR) and the echo time (TE), which can be independently adjusted to alter the magnetisation relaxation weighting of the image; number of echoes (if applicable) and the number of times the signal was averaged (NA). For most MRI experiments, two slices of the berry were imaged consecutively; one slice with a transverse orientation (through the centre of the berry, perpendicular to the central vascular bundles) and a second with a longitudinal orientation (through the centre of the berry, parallel to the central vascular bundles). The field of view (FOV) required adjustment for each berry imaged due to the variation in berry size. As a result, the voxels of each image differ in volume (as indicated in the figure captions), although all voxels had a volume less than 0.02 mm^3^. This is in contrast to clinical MRI scanners which have voxel volumes of 1 – 8 mm^3^.

A standard multi-shot multi-echo imaging (MSME) sequence (a CPMG based imaging sequence) was employed to produce two-dimensional images weighted by transverse relaxation. The sequence parameters used included THK 1 mm, MTX 256 × 256, a train of 16 echoes spaced 10 ms apart, TR 5 s and NA 2 (total acquisition time ~32 min).

A standard PGSE echo planar DTI sequence [29] was used to produce three-dimensional images weighted by diffusion. The sequence parameters used included THK 1 mm, MTX 128 × 128, *δ* 1 ms, Δ 25 – 50 ms, TE 35 – 65 ms, TR 6500 – 15000 ms and NA 1 (total acquisition time ~4.5 h). The Δ, TE and TR for the DTI sequences were adjusted for each age class of grape berry due to changes in the transverse and longitudinal relaxation properties of the berries across their development (Table 1). The TE was set in order to maximise the length of Δ, and minimise the loss of MR signal due to transverse relaxation. The TR was adjusted to be five times the length of the maximum longitudinal relaxation time (5 × *T*_1_) of each grape berry in order to minimise unintended longitudinal relaxation weighting. Two diffusion measurements (*b* weighting of 250 and 500 s mm^-2^) were performed along forty-two directions (the vertices of a pentakis icosidodecahedron). One *S*_0_ image (which had a negligible diffusion weighting) was also acquired in order to normalise the diffusion weighted images.

**Table 1:** **Summary of the Δ, TE and TR values used for the DTI sequences**

Berry age (DAF)	Δ (ms)	TE (ms)	TR (ms)
28	25	40	12500
41	50	65	15000
55	50	60	15000
70	50	60	10000
85	25	35	8000
95	25	35	7000
109	25	35	7000

These values were adjusted for each age class of grape berry due to changes in the transverse and longitudinal relaxation properties of the berries across their development.

**Figure 2:**
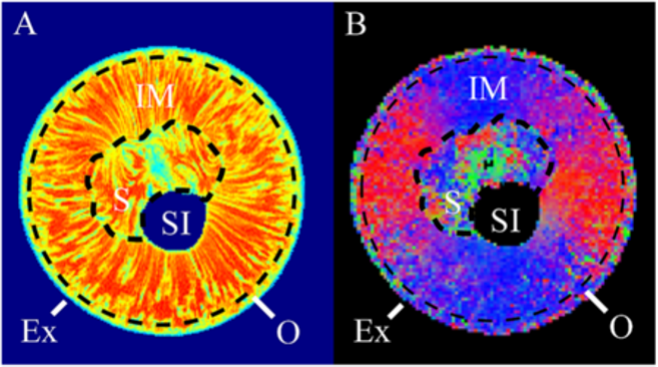
**Tissue regions of the grape berry (transverse plane).** Here the five tissue regions of the grape berry are provided with reference to a transverse relaxation image **(A)** and a diffusion tensor image **(B)**. Ex: exocarp, OM: outer mesocarp, IM: inner mesocarp, S: septum, SI: seed interior. The outer, black dashed curve indicates the border between the outer mesocarp and the inner mesocarp while the inner, black dashed curve indicates the border between the inner mesocarp and the septum.

For the analysis of the DT and transverse relaxation images, each grape berry was divided into four pericarp tissue groups (Figure 2); the exocarp, outer mesocarp, inner mesocarp and septum [30, 31]. The exocarp was defined as the outer epidermis and outer hypodermis tissue of the grape berry. The outer mesocarp was considered to be the tissue between the outer hypodermis and the tissue exterior to the peripheral vascular bundles, while the inner mesocarp was considered to be the tissue inwards from the peripheral vascular bundles. The septum was defined as the irregular tissue found at the centre of the grape berry adjacent to the berry seed(s) and locule(s). The endocarp was not considered in the analysis because it was beyond the resolution of the relaxation images. The seed interior was considered separately from the listed pericarp tissues. Each region in the images was analysed independently from all other regions by the application of image masks. These image masks were based on the tissue groups visible in the transverse relaxation images (in the manner explained previously).

For the analysis of the relaxation images, a mean *T*_2_ (i.e., spin-spin relaxation time) was determined for each of the pericarp tissues, from each grape berry in the same age group. A standard error was also calculated for each tissue based on the mean *T*_2_ values across each respective age group. The acquired DT image data, on the other hand, was used to create three images of each grape berry; a diffusivity map (a map of the average ADC for each voxel), a diffusion vector field map and a diffusion colour map (both of which indicate the direction of least restricted diffusion). The DT colour maps used a symmetrical additive red/blue/green colour scheme to represent that the direction of least restricted diffusion in each voxel was lateral/longitudinal/perpendicular with respect to the FOV. Mean diffusivity values and accompanying standard errors were calculated from the diffusivity maps on a per tissue basis in the same manner as the transverse relaxation maps.

A high spatial resolution DT image of a grape berry 55 DAF was used to confirm whether the radial striation patterns visible in the relaxation images were linked to variations in cellular size across the inner mesocarp tissue. This was done by using the *S*_0_ image (which has a small *T*_2_ weighting, and had visible striation patterns in the inner mesocarp region) to create an MR signal intensity threshold mask. The threshold value was qualitatively chosen in order to divide the inner mesocarp into radial bands of voxels with ‘relatively high’ and ‘relatively low’ signal intensity. The image mask was then applied to the DT image data to create two populations of voxels. The three eigenvalues of the DT (i.e. three orthogonal ADCs, the largest of which corresponds to the direction of least diffusion restriction) for each voxel were averaged over the two populations. This resulted in two groups of primary, secondary and tertiary eigenvalues. The mean primary eigenvalue was proportional to the average length of the cells in the voxel, while the mean secondary and tertiary eigenvalues corresponded to either the average width or depth of the cells. The Tukey-Kramer test (*P* =0.05) was then performed to determine whether differences between corresponding mean eigenvalues were statistically significant. A statistically significant difference between the values would indicate that there is a significant difference in average cell size across the bands of the inner mesocarp striation patterns.

A DT and transverse relaxation image of a postharvest olive (*Olea europaea* L., cv. Correggiolla) was also acquired in order to determine the applicability of diffusion MRI to fruits with high oil contents. The olive was stored and imaged under the same conditions as the grape berries. The transverse relaxation images were obtained using the MSME pulse sequence and parameters outlined above. The DT images, on the other hand, were acquired using a pulsed gradient stimulated echo (PGSTE) pulse sequence. The sequence parameters used included THK 1 mm, MTX 64 × 64, *δ* 6 ms, Δ 80 ms, TE 20 ms, TR 3500 ms and NA 4 (total acquisition time ~10 h). Two diffusion measurements (*b* weighting of 80000 and 160000 s mm^-2^) were performed along twenty directions (the vertices of a dodecahedron). One *S*_0_ image was also acquired in order to normalise the diffusion weighted images. For the analysis of the transverse relaxation and DT images of the olive, the pericarp was treated as a single tissue. This was due to the larger voxel size of the images and lack of distinctive tissue groups.

## Results

**Figure 3:**
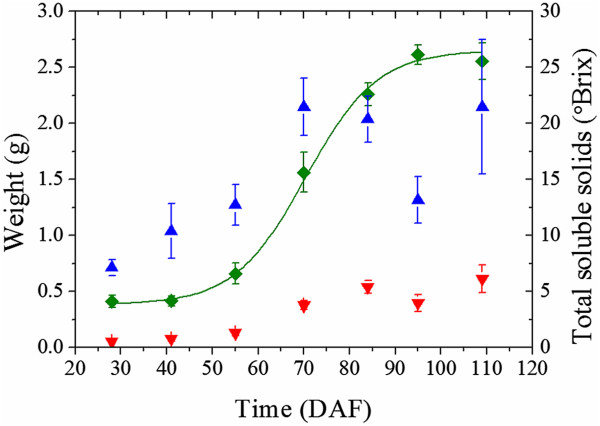
**The physical characteristics of the grape berries.** The concentrations of soluble solids of the grape berries (Green ♦), as well as the fresh weight (Blue ▲) and dry weights (Red ▼) of the berries, are presented with respect to the number of days after flowering. A sigmoidal function (solid green curve) of the form *a*_1_ + (*a*_2_ - *a*_1_)/(1 + exp(-(DAF - *x*_0_)/*w*)) was fitted to the soluble solids values by nonlinear regression (adjusted R^2^ = 0.99), where *a*_1_ = 26.1 (the approximate maximum soluble solids value), *a*_2_ = 3.9 (the approximate minimum soluble solids value), *x*_0_ = 69.7 (the inflection point) and *w* =7.4 (the change in DAF which yielded the greatest change in the soluble solids value). The error bars are given by the standard deviation of soluble solids values at each time point.

The grape berries increased in size and weight and then declined as the berries progressed through the ripening and senescence phases (Figure 3). The post-harvest olive had a fresh weight of 3.30 g and dry weight of 1.63 g. The concentration of soluble solids in the grape berries increased sigmoidally with respect to time (adjusted R^2^ = 0.99). Véraison occurred at approximately 60 – 65 DAF, and full ripeness was placed at 95 DAF (based on the mean concentration of soluble solids of the sampled berries which plateaued at 26 °Brix).

**Figure 4:**
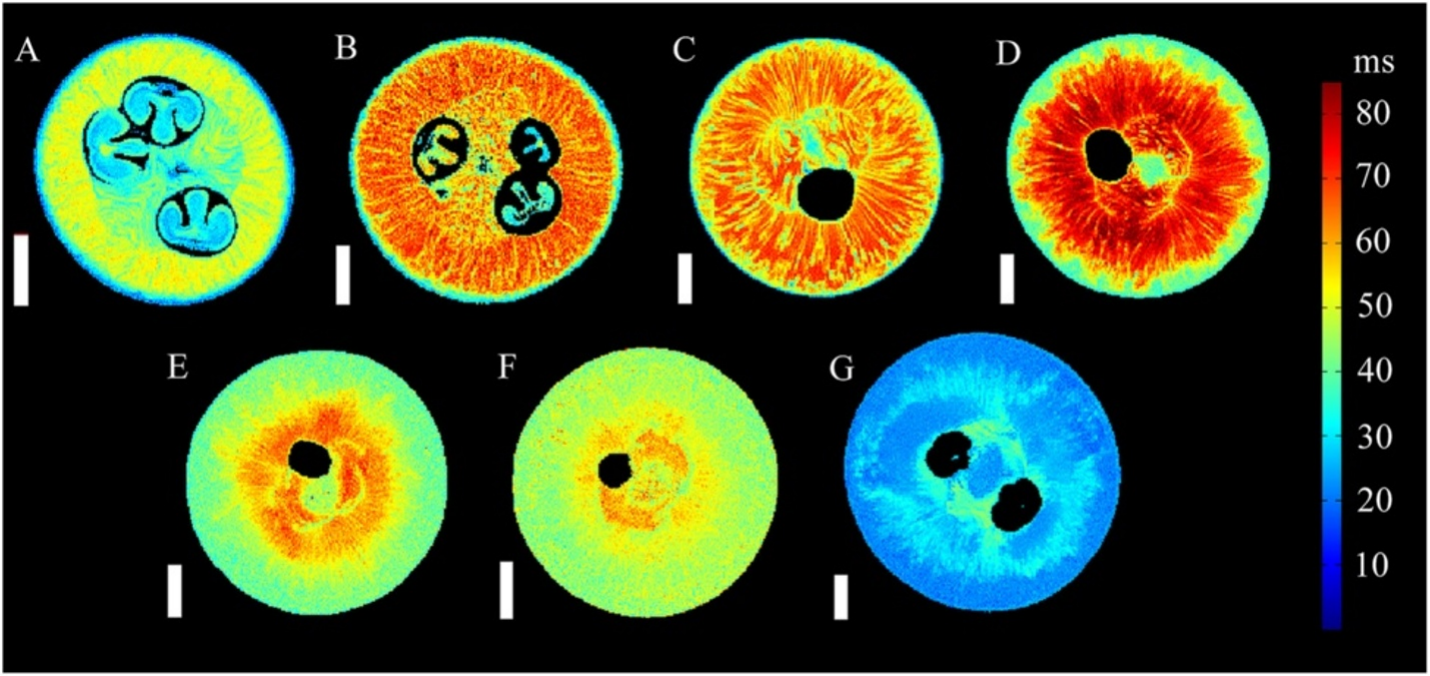
**Transverse relaxation images of grape berries at seven different stages of berry development (transverse plane).** The images include three pre-véraison grapes, at 28 DAF (**A**, voxel size 59 × 59 × 1000 μm), 41 DAF (**B**, voxel size 78 × 78 × 1000 μm) and 55 DAF (**C**, voxel size 78 × 78 × 1000 μm), a grape undergoing véraison at 70 DAF (**D**, voxel size 82 × 82 × 1000 μm), a ripening grape at 85 DAF (**E**, voxel size 74 × 74 × 1000 μm), a grape which is at oenological maturity at 95 DAF (**F**, voxel size 63 × 63× 1000 μm) and a post-maturity berry at 109 DAF (**G**, voxel size 86 × 86 × 1000 μm). The transverse relaxation values are indicated by the colour bar to the right of the figure. Scale bar: 3 mm.

**Figure 5:**
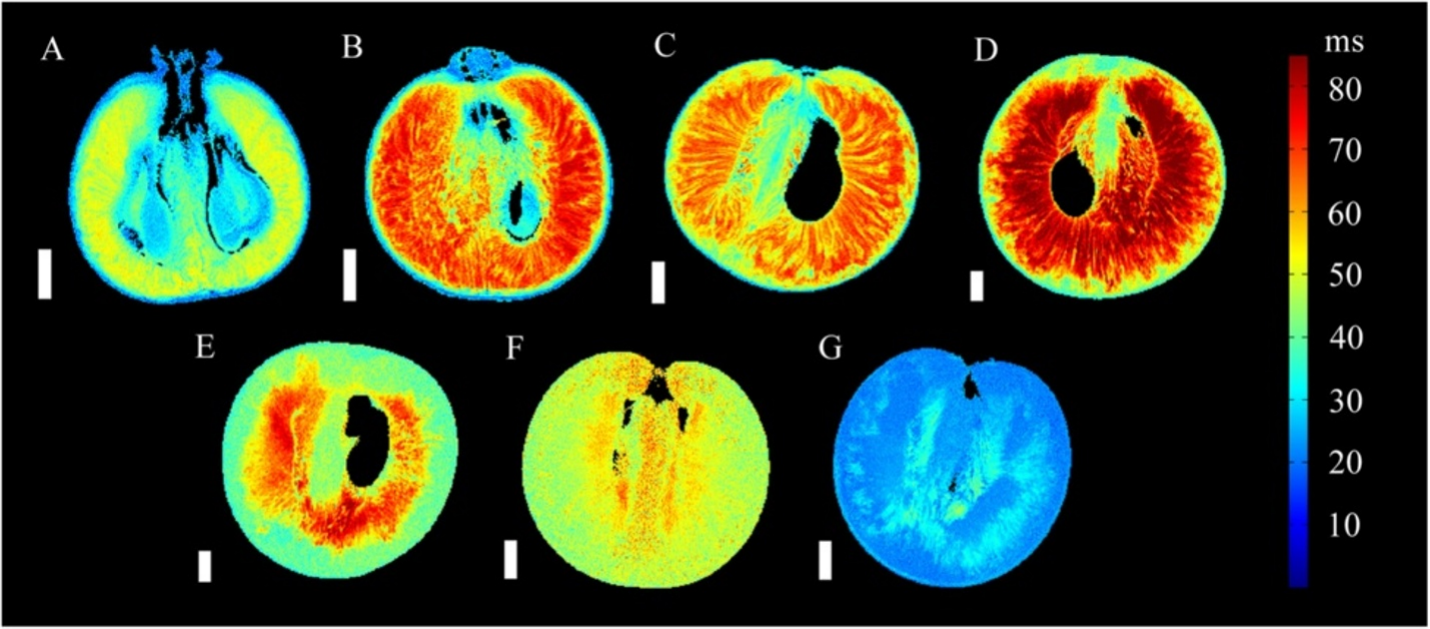
**Transverse relaxation images of grape berries at seven different stages of berry development (longitudinal plane)** ***.*** The images include three pre-véraison grapes, at 28 DAF (**A**, voxel size 59 × 59 × 1000 μm), 41 DAF (**B**, voxel size 78 × 78 × 1000 μm) and 55 DAF (**C**, voxel size 78 × 78 × 1000 μm), a grape undergoing véraison at 70 DAF (**D**, voxel size 133 × 133 × 1000 μm), a ripening grape at 85 DAF (**E**, voxel size 82 × 82 × 1000 μm), a grape which is at oenological maturity at 95 DAF (**F**, voxel size 63 × 63 × 1000 μm) and a post-maturity berry at 109 DAF (**G**, voxel size 86 × 86 × 1000 μm). The transverse relaxation values are indicated by the colour bar to the right of the figure. Scale bar: 3 mm.

**Figure 6:**
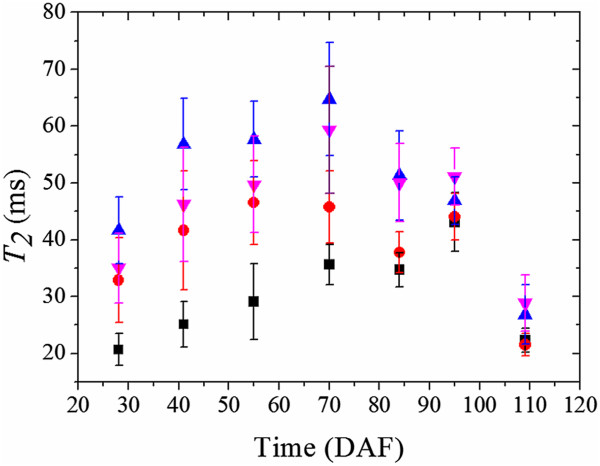
**Transverse (T**_**2**_ **) relaxation of the major tissue groups of the grape berry with respect to time (transverse plane).** The transverse relaxation values for the exocarp (■), outer mesocarp (Red ●), inner mesocarp (Blue ▲) and septum (Pink ▼). The error bars are given by the standard deviation of the transverse relaxation values at each time point.

**Figure 7:**
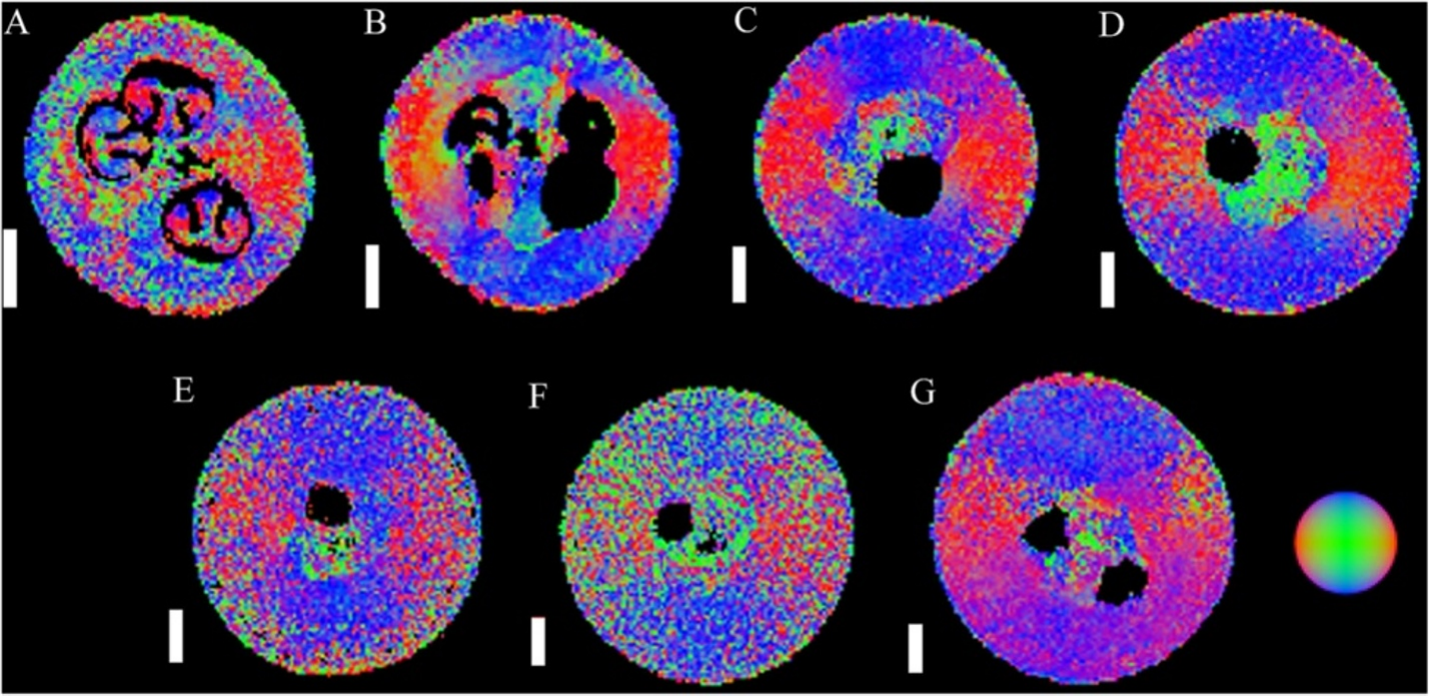
**DT images of grape berries at seven different stages of berry development (transverse plane).** The images include three pre-véraison grapes, at 28 DAF (**A**, voxel size 117 × 117 × 1000 μm), 41 DAF (**B**, voxel size 156 × 156 × 1000 μm) and 55 DAF (**C**, voxel size 156 × 156 × 1000 μm), a grape undergoing véraison at 70 DAF (**D**, voxel size 164 × 164 × 1000 μm), a ripening grape at 85 DAF (**E**, voxel size 148 × 148 × 1000 μm), a grape at oenological maturity at 95 DAF (**F**, voxel size 125 × 125 × 1000 μm) and a post-maturity berry, at 109 DAF (**G**, voxel size 171 × 171 × 1000 μm). The colours in the figure indicate the direction of least restricted diffusion, as indicated by the image at the bottom right of the figure. Scale bar: 3 mm.

**Figure 8:**
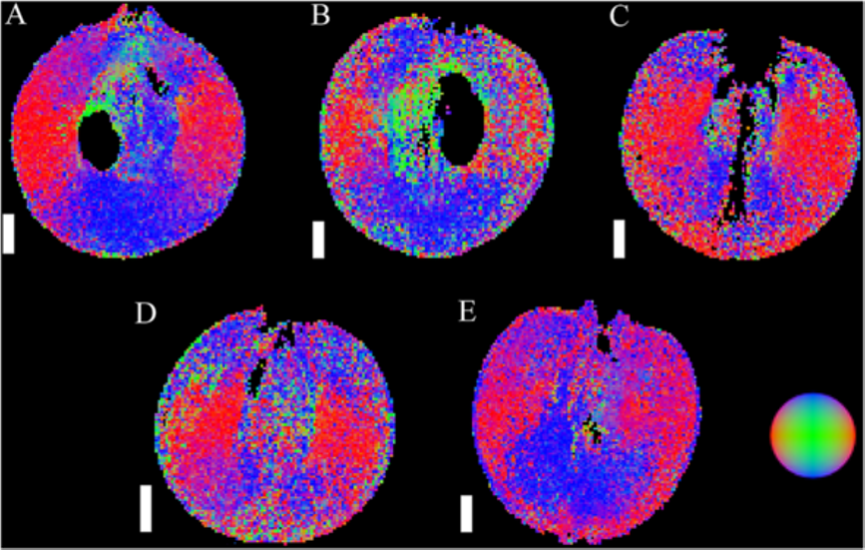
**DT images of grape berries at five different stages of berry development (longitudinal plane).** The images include a pre-véraison grape at 55 DAF (**A**, voxel size 156 × 156 × 1000 μm), a grape undergoing véraison at 70 DAF (**B**, voxel size 164 × 164 × 1000 μm), a ripening grape at 85 DAF (**C**, voxel size 172 × 172 × 1000 μm), a grape which is at oenological maturity at 95 DAF (**D**, voxel size 125 × 125 × 1000 μm) and a post-maturity berry at 109 DAF (**E**, voxel size 172 × 172 × 1000 μm). No images are available for 28 and 41 DAF. The colours in the figure indicate the direction of least restricted diffusion, as indicated by the image in the bottom right side of the figure. Images are not available for 28 and 41 DAF. Scale bar: 3 mm.

**Figure 9:**
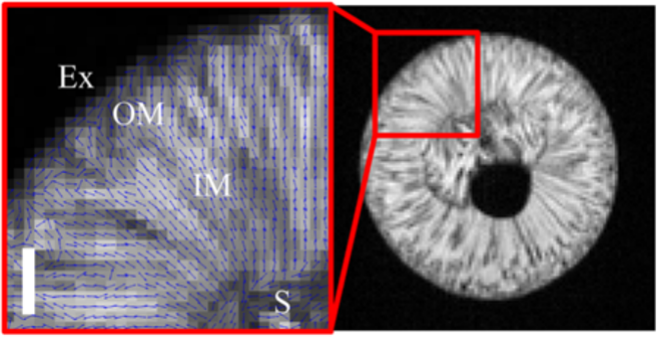
**Diffusion vector field map overlaying the S**_**0**_ **image of a grape berry 41 DAF (transverse plane).** The diffusion vectors (blue arrows) indicate the direction of least restricted diffusion in each voxel. Voxel size: 156 × 156 × 1000 μm, bar length: 1000 μm.

**Figure 10:**
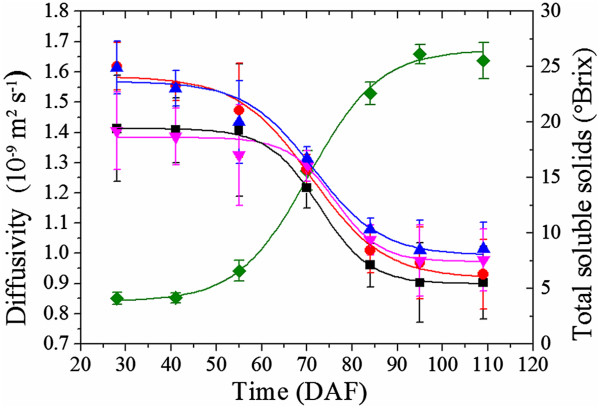
**Mean ADC of the major tissue groups of the grape berry with respect to time and total soluble solids** ***.*** The mean ADC for the exocarp (■), outer mesocarp (Red ●), inner mesocarp (Blue ▲) and septum (Pink ▼) decrease sigmoidally (adjusted R^2^ = 0.99) with respect to sigmoidally increasing (adjusted R^2^ = 0.99) dissolved solids content (Green ♦). The error bars are given by the standard deviation of the mean ADC at each time point.

In the transverse relaxation images, the exocarp was difficult to distinguish from the outer mesocarp (Figures 4 and 5). The exocarp was consistently associated with the shortest mean *T*_2_ at all sampled stages of grape berry development, relative to the other tissues examined (Figure 6). The diffusion vectors of the exocarp were prominently aligned tangential to the berry surface (Figures 7, 8 and 9). This pattern was consistent for the exocarp of all grape berries imaged, regardless of berry age. The exocarp was also found to have short mean diffusivity values relative to the other tissues (Figure 10).

The transverse relaxation images of the outer mesocarp generally lacked consistent or well defined features (Figures 4 and 5). The mean *T*_2_ of the outer-mesocarp tended to be longer than those of the exocarp, but shorter than other tissues (Figure 6). The diffusion vectors had a rotational dependency (Figures 7, 8 and 9) and were aligned so as to radiate from the centre of the fruit. In pre-véraison berries, the diffusivity values of the outer mesocarp were amongst the highest observed. For post-véraison grape berries, however, the mean diffusivity values declined further than other tissues (Figure 10).

**Figure 11:**
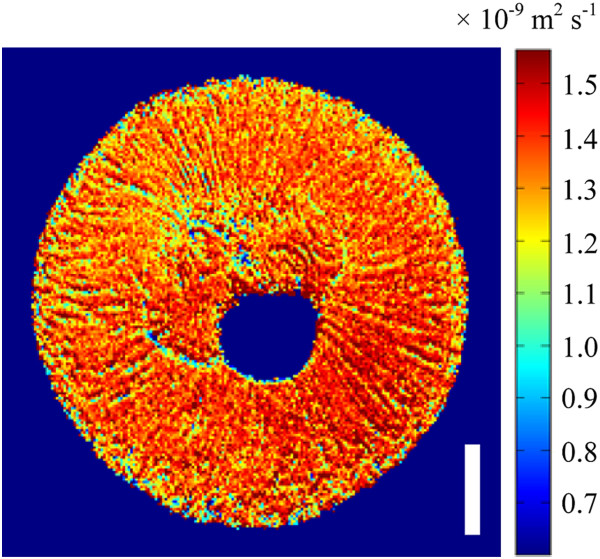
**Mean ADC map of a grape berry 55 DAF (transverse plane)** ***.*** Voxel size 78 × 78 × 1000 μm, bar length: 3000 μm.

**Figure 12:**
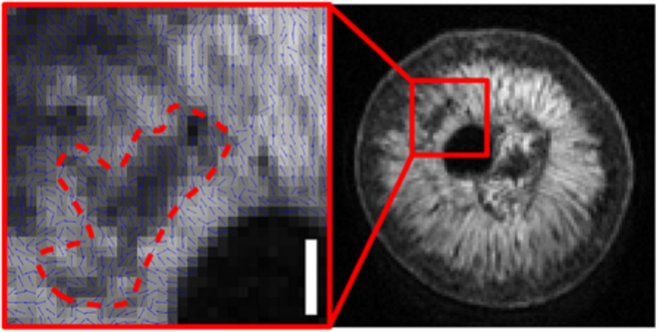
**Diffusion vector field map overlaying the S**_**0**_ **image of a grape berry 109 DAF (transverse plane).** The orientation of diffusion vectors are indicated by the blue arrows. There was a loss of diffusion-weighted signal in the region denoted by the red dashed line. Voxel size: 133 × 133 × 1000 μm, bar length: 1000 μm.

A notable feature in the transverse relaxation images of the inner mesocarp was the clear radial striation patterns with distinctly different (Tukey-Kramer test, *P* =0.05) *T*_2_ values. These patterns were observed in both transverse and longitudinal image orientations for the inner mesocarp (Figures 4 and 5) of grape berries between 28 DAF and 109 DAF. The striation pattern radiated throughout the entire sub-tissue, starting close to the septum and vascular systems at the centre of the berry (i.e., at the ovular and axial vascular network), and terminating at the interface between the inner and outer mesocarp. In addition, upon analysing the high resolution DT image of a grape berry 55 DAF (Figure 11), a statistically significant difference (Tukey-Kramer test, P =0.05) was noted between the mean secondary and tertiary eigenvalues across the striation bands (Table 2). Prior to 95 DAF, the mean *T*_2_ of the inner mesocarp was consistently higher than other tissues (Figure 6). After the concentration of soluble solids plateaued, the mean *T*_2_ of this tissue declined, accompanied by a partial loss of its radial striation pattern. The diffusion vectors of the inner mesocarp had a rotational dependency similar to that of the outer mesocarp. Furthermore, the diffusion vectors were predominantly parallel to the radial striation bands (Figures 7, 8 and 9), except for grape berries 28 DAF or past 95 DAF (Figure 12). After véraison, the inner mesocarp was also consistently associated with the largest mean diffusivity values, relative to the other tissues examined (Figure 10).

**Table 2:** **Comparing the mean eigenvalues of diffusion tensors associated with different relaxation striation bands**

	Mean primary eigenvalue (×10 ^-9^ m ^2^ s ^-1^)	Mean secondary eigenvalue (×10 ^-9^ m ^2^ s ^-1^)	Mean tertiary eigenvalue (×10 ^-9^ m ^2^ s ^-1^)
**Striation bands with high signal intensity**	1.53*	1.44	1.36
**Striation bands with low signal intensity**	1.54*	1.41	1.30

The standard error was not included as it was insignificant (<1% of the values listed). * : values between which there was no statistically significant difference (Tukey-Kramer test, *P* =0.05).

The septum was readily distinguishable from the surrounding mesocarp tissue in the transverse relaxation images. This was due to the presence of curved striation patterns which were perpendicular to the striation patterns of the inner mesocarp (Figures 4 and 5). These striation patterns were curled about the central vascular bundles and extended through the septum to the seeds and locules. Prior to 95 DAF, the mean *T*_2_ of the septum was consistently higher than the exocarp, but lower than the inner mesocarp. After 95 DAF, the septum had the highest mean *T*_2_ (Figure 6). Unlike the inner mesocarp, the diffusion vectors of the septum were not consistently parallel to the striation patterns of the tissue (Figures 7, 8 and 9). The anisotropic diffusion pattern of the septum differed on a per berry basis. The septal-mesocarp boundary could be discerned by abrupt changes in the local orientation of diffusion vectors. The mean diffusivity value of the septum was the lowest of all the examined tissues pre-véraison, but had a mean diffusivity greater than that of the exocarp and outer mesocarp post-véraison (Figure 10).

**Figure 13:**
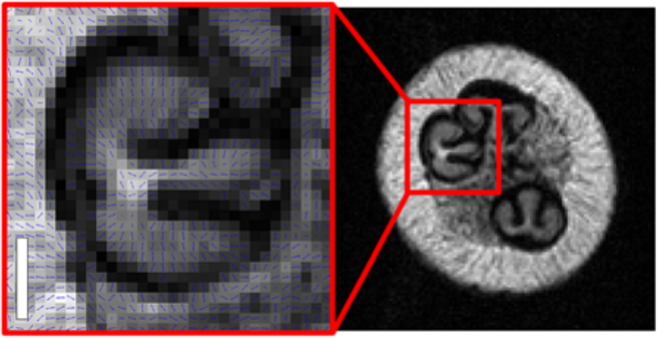
**Diffusion vector field map overlaying the S**_**0**_ **image of a grape berry seed interior 28 DAF (transverse plane)** ***.*** The diffusion vectors (blue arrows) indicate the direction of least restricted diffusion. Voxel size: 117 × 117 × 1000 μm, bar length: 1000 μm.

In the grape seed interior, the nucellus (located towards the bulbous distal end of the seed) was identified in grape berries aged 28 and 41 DAF. It could be readily identified in berries of this age, as it occupies a sizeable portion of the seed and has a stylised ‘3’ shape when imaged through the transverse plane [4]. The nucellus demonstrated a highly characteristic anisotropic diffusion pattern which was rotationally dependent, similar to the anisotropic diffusion pattern of the inner mesocarp tissue (Figures 7, 8 and 13). After 41 DAF, the MR signal from this region decayed too rapidly to be visible in the DT and transverse relaxation images.

**Figure 14:**
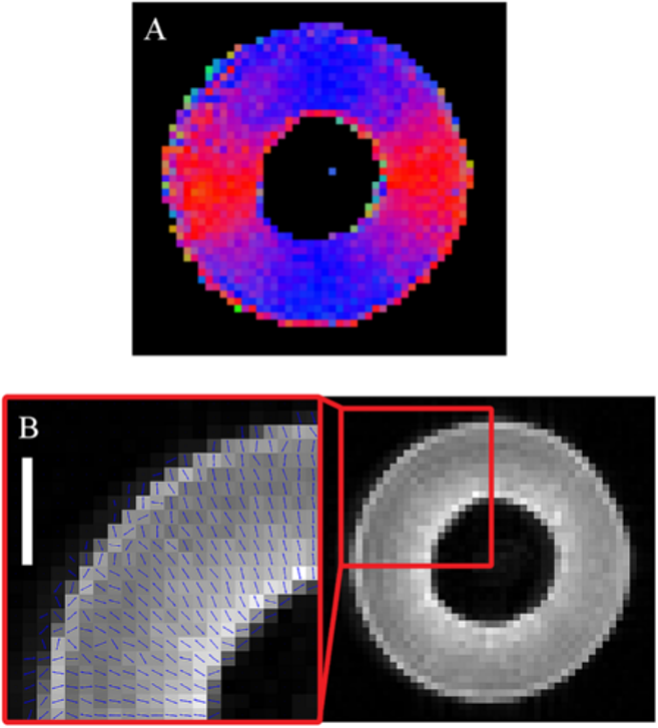
**DT and transverse relaxation images of a postharvest olive (transverse plane).** The DT image (**A**, voxel size 390 × 390 × 1000 μm) and diffusion vector map (**B**, voxel size 390 × 390 × 1000 μm) of an olive pericarp. Bar length 1000 μm.

The transverse relaxation images of the postharvest olive pericarp lacked well-defined macroscopic features, such as the radial striation patterns noted in the mesocarp of the grape berries. However, the anisotropic diffusion patterns (Figure 14A) and diffusion vectors (Figure 14B) associated with the olive pericarp exhibited a rotational dependency and were aligned so as to radiate from the centre of the fruit. The mean *T*_2_ of the olive pericarp was 45 ± 3 ms while the mean diffusivity value of the olive pericarp was 6.07 ± 0.46 × 10^-12^ m^2^ s^-1^.

## Discussion

**Figure 15:**
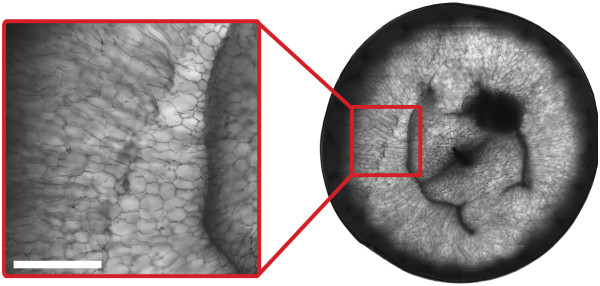
**Confocal micrograph of the pericarp of a grape berry prior to véraison 41 DAF (transverse plane).** The image was acquired using a confocal microscope (LSM5 Pascal; Zeiss, Germany) which employed a 488 nm Argon laser and a 10 × objective Plan-Apochromatic lens. Bar length 1000 μm.

**Figure 16:**
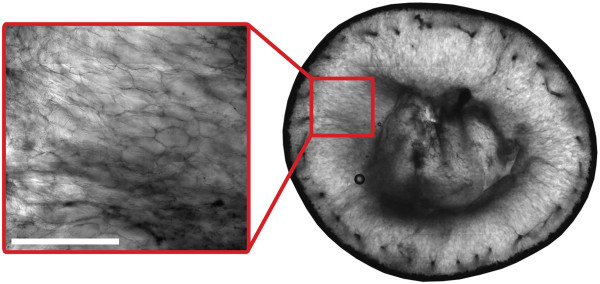
**Confocal micrograph of the pericarp of a grape berry undergoing véraison 55 DAF (transverse plane).** The image was acquired using a confocal microscope (LSM5 Pascal; Zeiss, Germany) which employed a 488 nm Argon laser and a 10 × objective Plan-Apochromatic lens. Bar length 1000 μm.

The anisotropic diffusion patterns observed in the grape berry pericarp were due to the restricting effects of cell membranes on diffusion. For example, in the mesocarp of grape berries 28 DAF, the diffusion anisotropy exhibited low coherence. This was because the parenchyma cells of the mesocarp were not fully elongated [32], as demonstrated by confocal microscopy (Figure 15). Between 41 DAF and 95 DAF, however, the anisotropic diffusion pattern was clearly radially dependent, thus reflecting the radial orientations of the elongated inner mesocarp cells [3, 30, 32], as shown by confocal microscopy (Figure 16). The orientations of the diffusion vectors in the inner mesocarp were also preferentially parallel to the radial striation patterns visible in the transverse relaxation images, a relationship that will be discussed further below.

Upon approaching full ripeness, regions of the berry inner mesocarp exhibited a loss of coherence in orientation of diffusion anisotropy (Figure 12). This was accompanied by a decline in *T*_2_ values and partial loss of the radial striation pattern in the transverse relaxation images of these same regions. The decline of *T*_2_ values would begin at the interface between the outer and inner mesocarp, and would steadily shift toward the centre of berry as the grape approached the fully ripened stage. Between 85 and 109 DAF, the *T*_2_ values for all berry tissues noticeably decreased. An increase in cellular fluid viscosity could reduce the rotational velocity of spin bearing molecules in the berry; however, there is no straightforward relationship between the observed transverse relaxation rate and the measured concentration of soluble solids in the final stages of berry ripening. It is also doubtful that the increased transverse relaxation rate could be caused by a sudden influx of free paramagnetic ions. Due to the small width of the voxels constituting the *T*_2_ images (i.e. 60 – 80 μm) the read gradients of the *T*_2_ MR imaging sequence may have caused unaccounted diffusion-weighted signal relaxation [33]. However, this seems unlikely, as *T*_2_ MR images have previously been taken of a geranium leaf petiole with a voxel width of 39 μm [34]. Despite the small voxel width, these images exhibited a strong contrast between the primary tissues of the petiole. The maximum observed *T*_2_ value of the petiole was also less than 85 ms, similar to the maximum observed *T*_2_ values of the grape berries. It is possible that the observed loss of diffusion vector coherence and the decrease in *T*_2_ values is linked to widespread cell death occurring throughout the berry mesocarp. This phenomenon is known to occur across the mesocarp of certain wine grape berries during the later stages of ripening [35, 36]. Although Semillon grape berries were not included in these studies, for the varieties tested cellular vitality began to decrease at a point between 85 and 109 DAF.

The radial striation patterns noted in the grape berry mesocarp were also noted in a previous investigation [3]. Bright field light micrographs of the berry inner mesocarp indicated that these patterns arise in the MR images due to the arrangement of alternately sized radially elongated parenchyma cells in the grape berry mesocarp tissue. The results of the current study agree with this theory. There was no statistically significant difference between mean primary eigenvalues from striation bands with ‘relatively high’ and ‘relatively low’ MR signal intensity, thus indicating that the cells belonging to both populations have the same mean length. However, there was a statistically significant difference between the mean secondary and tertiary eigenvalues of the two populations. The ‘relatively low’ MR signal intensity population was associated with the lower eigenvalues, indicating that the cells belonging to this population were less wide than their counterparts in the ‘relatively high’ MR signal intensity population. As the parenchyma cells are radially elongated in grape berries older than 28 DAF, the transverse relaxation striation patterns thus aligned with the anisotropic diffusion patterns in the mesocarp of these berries. While the transverse relaxation images of the septum demonstrated striation patterns similar to those found in the inner mesocarp, the orientation of anisotropic diffusion within the septum was largely not aligned to these septal striation bands. The lack of correlation between the septal striation patterns and diffusion anisotropy is likely due to the irregular size and shape of the septal cells [30].

The rotationally dependent anisotropic diffusion patterns noted in the outer edge of young seed nucelli are due to the diffusion of water through the seed integument. These cells are known to be tabular in shape and are radially arranged [37]. However, after 41 DAF, there was a decline in the measurable MR signal from the integument tissue due to tissue dehydration and mechanical hardening. Concurrently, the seed nucellus deteriorated and was replaced by the liquid endosperm [38, 39]. The liquid endosperm has a transverse relaxation significantly less than 30 ms (~10 ms). As a result, after 41 DAF the MR signal from the seed interior decayed too rapidly for the imaging protocols used in the current study.

### Comparing the results of the study to the literature

The correlation observed in the present study between anisotropic diffusion patterns and the known structure of grape berry pericarp tissues was in contrast to the recent DTI study of grape berries by Gruwel et al., [13]. While the experimental procedures employed in both studies were largely similar, they differed in two important respects. First, the TE of the PGSE MRI pulse sequence used in the previous study was shorter than those used in the current study. TE limits the maximum length of Δ, so although the length of Δ was not specified in the study by Gruwel et al., it can be inferred from the TE used (26 ms) that it was less than the shortest Δ employed in the current study. This difference is noteworthy because as Δ increases, the water molecules have more time to displace further from their origin and interact with different physical structures. The cells of the berry vasculature are far smaller than the cells that make up the mesocarp, thus a short Δ relative to that used in the current study remains appropriate for probing the cellular membranes of the xylem and phloem. However, in regions where the average cell size is much larger, (e.g. the inner mesocarp) the diffusion anisotropy patterns are more likely to be observed due to interactions with cellular components rather than the cellular membrane. As a result, the observations from both the current study and that of Gruwel et al. could be considered accurate; however, they are observations of the effects of different restricting structures (i.e., on a shorter length scale).

The second difference between the present study and that of Gruwel et al. was the diffusion gradient schemes employed. Gruwel et al. performed diffusion measurements along six unique diffusion gradient vectors. While this is the minimum required for DTI, it is possible that gradient cross-terms (from the imaging and diffusion gradients) have affected the diffusion weighting of the images. The current study used forty-two different diffusion gradient vectors (i.e., twenty-one unique directions and their corresponding opposite). The inclusion of gradient vectors and their corresponding opposite gradient vectors reduces the effects of potential gradient cross-terms [27, 40]. Using a large number of unique diffusion gradient vectors also reduces potential directional bias when reconstructing **D**.

### The applicability of diffusion MRI to plant tissues differing in oil and water content

The question remains as to the applicability of diffusion MRI in the examination of other plant species and organs. This will chiefly depend upon the amount of unbound water the organ contains. Tissues with low content, such as the solid endosperm of seeds, are difficult to examine. In addition, tissues with smaller cells are easier to examine than tissues with larger cells. However, providing that Δ is long enough to ensure that a large population of water molecules are able to interact with the surface of the cellular boundaries (e.g. Δ ~ *d*^2^/*D* where *d* is the distance between cellular boundaries), diffusion anisotropy will be evident.

The applicability of diffusion MRI to the study of plant species with high oil contents is of great interest; oil is hydrophobic and thus diffusing water molecules are likely to treat any droplets of oil encountered in cell cytoplasm [41] as diffusion restricting obstacles. Depending on the concentration of oil in the cytoplasm, water diffusivity could be noticeably affected. Olives are an ideal candidate for such a study as they are comparable in size to grape berries and also have a similar internal tissue structure and cellular dimensions. These features would aid comparisons to the results of the current study. To determine the practicality of such an investigation, a postharvest olive was imaged using both DT and transverse relaxation MRI.

Due a noticeable loss of radio-frequency magnetic pulse power, fast diffusion MRI sequences could not be used, increasing the length of the total image acquisition. This was a consequence of the high salt content of the fruit. The low mean diffusivity value of olive pericarp also necessitated the use of very high diffusion magnetic gradients to perform the diffusion measurements. A lengthy diffusion period was also required to ensure that the slowly diffusing molecules had sufficient time to probe the restricting tissue microstructure. To prevent prohibitive MR signal loss arising from transverse relaxation, a PGSTE MRI sequence was employed. While the two pulse sequences measure diffusion in the same way, the PGSTE sequence halves the intensity of the acquired MR signal. The number of image averages and the size of the image voxel were thus increased to compensate for this loss of signal.

The resultant diffusion MRI images had voxel volume less than 1 mm^3^ and a good signal-to-noise ratio. The radial anisotropic diffusion pattern observed in the olive pericarp was similar to the grape berries close to or past véraison, indicating that the cells of the mesocarp are likewise radially elongated. This result agrees with what is known of the olive mesocarp structure [42]. The mean *T*_2_ value of the olive was comparable to those of the grape berry pericarp tissues, as was expected for fruits with similar cellular dimensions. The large difference between the mean ADC of the olive pericarp and the grape berry pericarp tissues is likely due to the presence of oil in the cell cytoplasm. However a more in-depth investigation is required to speculate further.

## Conclusion

The results of the current study have demonstrated that diffusion anisotropy patterns are correlated with the microstructure of the major pericarp tissues of cv. Semillon grape berries, including the exocarp, outer and inner mesocarp, and seed interior. Changes in grape berry tissue structure during berry development, which were observable in the DT and transverse relaxation images, have also been described. These changes in tissue structure included the transition from non-elongated to radially elongated mesocarp cells between 28 and 41 DAF, which was associated with an increase in diffusion vector coherence. Conversely, from the mid-ripe phase and beyond, the loss of diffusion vector coherence was possibly the result of the widespread cell death in the mesocarp that often accompanies this stage of development in wine grapes. Rotationally dependent anisotropic diffusion patterns were also noted in the seed integument of young grape berries. These diffusion patterns could only be observed early in the development of the grape berry, due to the dehydration of the seed integument and the replacement of the nucellus by the liquid endosperm.

The results of the current study agree that the inner mesocarp striation patterns previously noted in the transverse relaxation images of previous studies arise due to variations in cell width across the striation bands. These bands were evident in the images of the grape berries at 28 DAF and largely persisted through all subsequent phases of berry development.

## Abbreviations

ADC: Apparent diffusion coefficient
CPMG: Carr-Purcell-Meiboom-Gill
DAF: Days after flowering
DT: Diffusion tensor
DTI: Diffusion tensor imaging
FOV: Field of view
MTX: Matrix size
MR: Magnetic resonance
MRI: Magnetic resonance imaging
NA: Number of averages
PGSE: Pulsed gradient spin-echo
RMSD: Root mean square displacement
TE: Echo time
THK: Slice thickness
TR: Repetition time
*T*_1_: Longitudinal relaxation time
*T*_2_: Transverse relaxation time
*δ*: Duration of the applied magnetic field gradient pulse
Δ: Timescale of the diffusion measurement.
