# Erratum to: Use of diffusion magnetic resonance imaging to correlate the developmental changes in grape berry tissue structure with water diffusion patterns

**DOI:** 10.1186/s13007-016-0106-x

**Published:** 2016-01-27

**Authors:** Ryan J. Dean, Timothy Stait-Gardner, Simon J. Clarke, Suzy Y. Rogiers, Gabriele Bobek, William S. Price

**Affiliations:** Nanoscale Organisation and Dynamics Group, University of Western Sydney, Penrith, NSW 2751 Australia; National Wine & Grape Industry Centre, Charles Sturt University, Locked Bag 588, Wagga Wagga, NSW 2678 Australia; New South Wales Department of Primary Industries, Locked Bag 588, Wagga Wagga, NSW 2678 Australia; School of Medicine, University of Western Sydney, Penrith, NSW 2751 Australia

## Erratum to: Plant Methods 2014, 10:35 DOI 10.1186/1746-4811-10-35

Unfortunately, the original version of this article [1] contained an error in Figure 8. In this figure the sub-images have been placed in the wrong order and do not match the corresponding figure legend. The correct version of Fig. 8 can be found below.

**Fig. 8:**
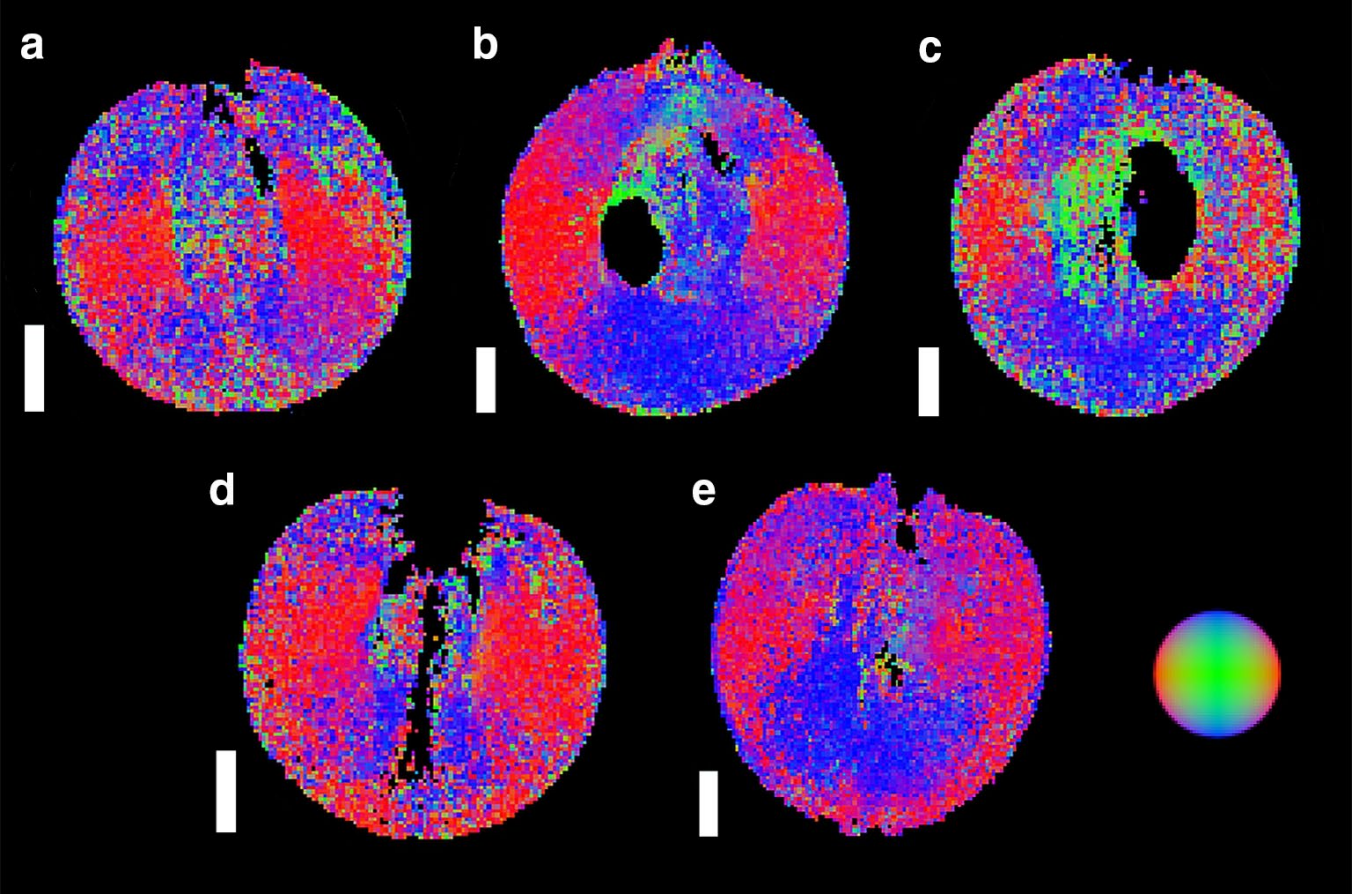
DT images of grape berries at five different stages of berry development (*longitudinal plane*). The *images* include a pre-véraison grape at 55 DAF (**a**, voxel size 156 × 156 × 1000 μm), a grape undergoing véraison at 70 DAF (**b**, voxel size 164 × 164 × 1000 μm), a ripening grape at 85 DAF (**c**, voxel size 172 × 172 × 1000 μm), a grape which is at oenological maturity at 95 DAF (**d**, voxel size 125 × 125 × 1000 μm) and a post-maturity berry at 109 DAF (**e**, voxel size 172 × 172 × 1000 μm). No images are available for 28 and 41 DAF. The *colours in the figure* indicate the direction of least restricted diffusion, as indicated by the image in the bottom right side of the figure. Images are not available for 28 and 41 DAF. *Scale bar* 3 mm
